# Characterization of transcriptome diversity and in vitro behavior of primary human high-risk breast cells

**DOI:** 10.1038/s41598-022-10246-4

**Published:** 2022-04-22

**Authors:** Sahar J. Alothman, Keunsoo Kang, Xuefeng Liu, Ewa Krawczyk, Redha I. Azhar, Rong Hu, David Goerlitz, Bhaskar V. Kallakury, Priscilla A. Furth

**Affiliations:** 1 Department of Oncology, Georgetown University, 3970 Reservoir Rd NW, Washington, DC 20057 USA; 2 Department of Microbiology, College of Science and Technology, Dankook University, Cheonan, 31116 Republic of Korea; 3 Department of Pathology, Georgetown University, Washington, DC 20057 USA; 4 Center for Cell Reprogramming, Georgetown University Medical Center, Washington, DC USA; 5 Department of Medicine, Georgetown University, Washington, DC 20057 USA

**Keywords:** Cancer, Genetics

## Abstract

Biology and transcriptomes of non-cancerous human mammary epithelial cells at risk for breast cancer development were explored following primary isolation utilizing conditional reprogramming cell technology from mastectomy tissue ipsilateral to invasive breast cancer. Cultures demonstrated consistent categorizable behaviors. Relative viability and mammosphere formation differed between samples but were stable across three different mammary-specific media. E2F cell cycle target genes expression levels were positively correlated with viability and advancing age was inversely associated. Estrogen growth response was associated with Tissue necrosis factor signaling and Interferon alpha response gene enrichment. Neoadjuvant chemotherapy exposure significantly altered transcriptomes, shifting them towards expression of genes linked to mammary stem cell formation. Breast cancer prognostic signature sets include genes that in normal development are limited to specific stages of pregnancy or the menstrual cycle. Sample transcriptomes were queried for stage specific gene expression patterns. All cancer samples and a portion of high-risk samples showed overlapping stages reflective of abnormal gene expression patterns, while other high-risk samples exhibited more stage specific patterns. In conclusion, at-risk cells preserve behavioral and transcriptome diversity that could reflect different risk profiles. It is possible that prognostic platforms analogous to those used for breast cancer could be developed for high-risk mammary cells.

## Introduction

Breast cancer is a heterogeneous disease with contributions from different pathophysiologic mechanisms including lifestyle, environment, physiological and genetic factors^1,2^. Late breast cancer recurrence estimates vary but range around 15% with hormone positive cancers being the most likely to recur^3^. A better understanding of underlying biology has been proposed as a means to improve individual risk calculation for development of secondary breast cancers^4^. Breast cancer exhibits field cancerization, meaning that normal-appearing breast cells ipsilateral to a known cancer are at higher risk than normal for development into a secondary breast cancer^5^. Risk factors predisposing an individual to breast cancer may also be related to recurrence of breast cancer. For example, normal developmental programs that go awry can contribute to breast cancer formation^6^. Expansion and differentiation of normal mammary stem cells with mammary gland transitions induced by hormonal cycling such as the menstrual cycle and pregnancy may also serve to generate cancer precursor stem cells^7–9^. Previously, menstrual cycle induced gene expression changes in women were defined from dissected mammary epithelium by bulk RNA sequencing^9^ and single-cell RNA profiling used to develop a cellular blueprint of normal human breast epithelium^10^. Because mice and humans share mammary gland developmental gene expression patterns during pregnancy^11^, transcriptional profiles developed using mice^8^ can provide a comparator for changes in gene expression that are more difficult to characterize using human tissue. Reduction mammoplasty tissue has been used to define age associated changes in gene expression^12^ and ductal lavage cells for transcriptome assessment of individuals at high versus normal breast cancer risk^13^. Several transcriptome-based breast cancer prognostic platforms are validated through clinical trials^14^, but profiles for breast cancer risk prediction are not yet fully realized^3,4,15,16^. One goal of the present study was to assess diversity and pattern formation within transcriptional profiles of human high-risk mammary cells utilizing RNAseq.

A second goal was to address the biology of high-risk human mammary epithelial cells. A pillar of cancer-related biological investigations are cell culture models^17^. In this study we focused on primary cells, more immediately reflective of in vivo disease in people, to address our questions. While primary epithelial cells can be challenging to acquire, we utilized a time and cost-effective method, the epithelial-specific conditionally reprogrammed cells (CRC) technique, for efficient isolation. This technique enables maintenance of intact gene expression profiles through early passage^18–21^. It can be used in its original formulation with a fibroblast feeder layer or as CRC conditioned media (CRC^CM^), enabling culture without the fibroblast feeder layer^20^. CRC-isolated mammary cultures have been reported to maintain expression of Estrogen Receptor 1 (*ESR1*) through early passage, a known challenge in studying the biology of mammary epithelial cells^22,23^. Because maintenance of *ESR1* expression in vitro is also facilitated by growth as 3D mammospheres^24^, we chose matrix-free scaffold based nano-culture plates permitting both 2D monolayer and 3D sphere growth for a relatively high throughput analysis^25^. Cultures were moved into a secondary media lacking both phenol-red and serum for hormonal testing^26,16^. Three commercially available mammary-specific media are available, each developed for different purposes. Serum-free Mammary Epithelial Growth Medium (MEGM) (Lonza, Walkersville, MD, USA) maintains primary mammary epithelial cell heterogeneity through early passage. EpiCult™ and MammoCult™ Mammary Cell Culture Media (Stemcell Technologies, Vancouver, CA) support monolayer mammary precursor cell versus mammosphere growth, respectively^19,27^. Parallel replicative testing in each media was performed to assess within and between sample stability of viability and mammosphere formation in different medium to judge if viability and mammosphere formation were intrinsic to each sample or imposed by the medium. Because neither CRC^CM^ or any of the mammary specific media had been evaluated in conjunction with the matrix-free scaffold-based nano-culture plates, this was considered an essential variable to appraise for interpretation of sample differences. Mycoplasma infection of the primary cultures was evaluated by both biochemical testing and screening of the RNAseq data for mycoplasma RNA sequences^28,29^.

Transcriptional profiling contributes to molecular understanding and provides leads for mechanistic investigations^30^. Because our study combined transcriptional profiling with biological behavior in primary cell culture, we utilized Gene Set Enrichment Analysis (GSEA)^31^ and the Molecular Signatures Database^32,33^ for associations and patterns between gene expression and behavior. Previous research shows that individual gene expression patterns can be linked to specific cell behaviors including viability^34–39^ and estrogen response^40,41^.

## Results

### Relative viability and mammosphere formation were preserved properties across different media conditions inherent to individual samples

**Figure 1:**
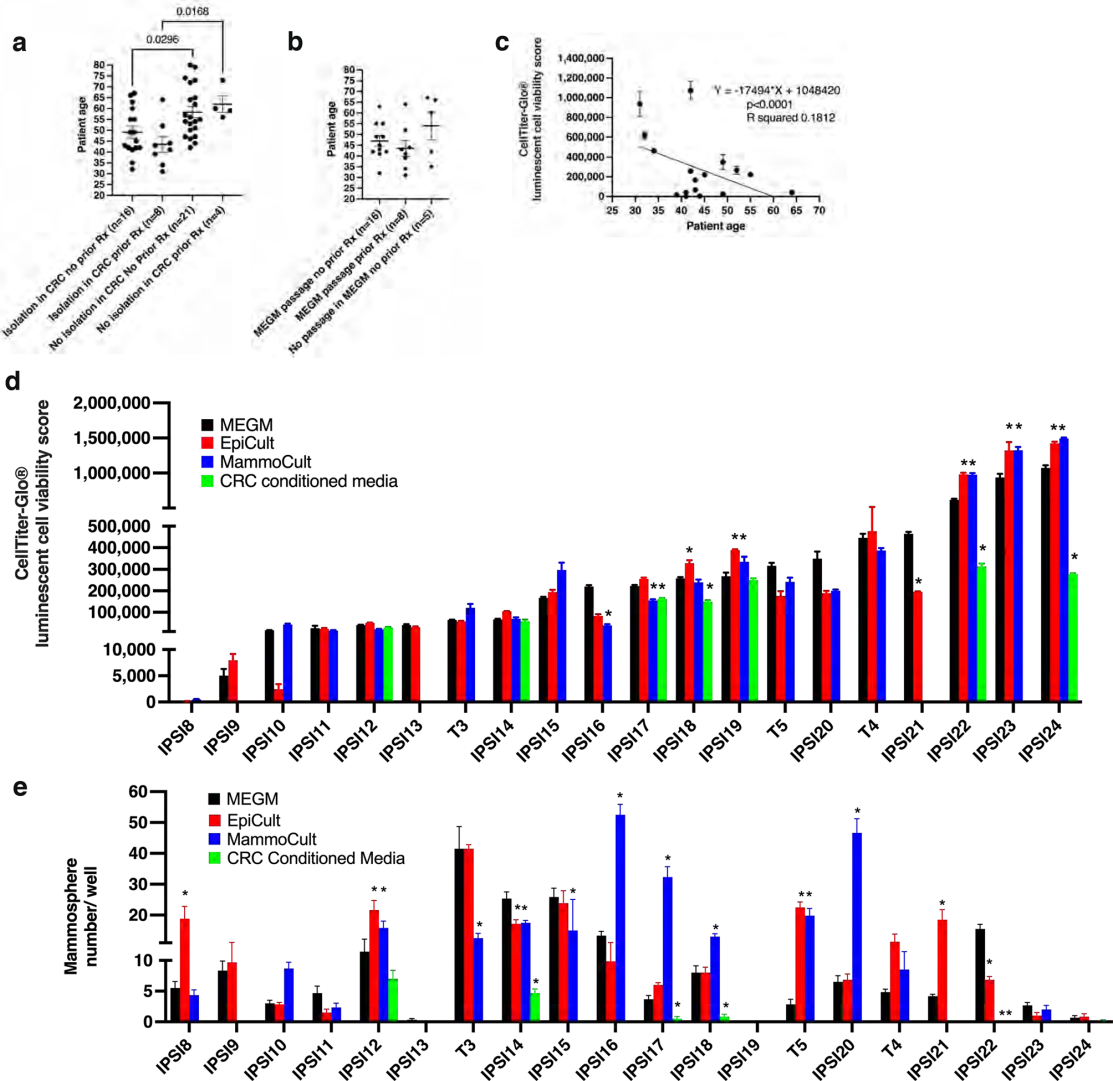
Viability and mammosphere formation in different media. (**a**) Scatter plot illustrating age distribution of samples isolated and not isolated in CRC with and without prior neoadjuvant therapy (Rx). Sample numbers, mean and standard error of the mean (SEM), and *p* values < 0.05 shown. Ordinary one-way ANOVA, *p* = 0.0035 F = 5.218, Brown-Forsythe test F = 0.6113, DFn = 3 DFd = 45, Sidak’s multiple comparisons test padj = 0.0296 [Isolation in CRC No prior Rx n = 20, No isolation in CRC No prior Rx n = 21], padj = 0.0168 [Isolation in CRC Prior Rx n = 7, No isolation in CRC Prior Rx n = 4]. (**b)** Scatter plot illustrating age distribution of samples passaged and not passaged in MEGM with and without prior neoadjuvant therapy (Rx). Sample numbers and mean and SEM shown. (**c**) Regression scatter plot: patient age (years) and viability measured in MEGM. n = 6 replicates. Regression equation, *p* value and R squared shown. (**d)** Bar graphs presenting cell viability of primary cells measured seven days following plating in different media (n = 6 replicates/condition with exceptions IPSI9 n = 3 replicates/EpiCult, IPSI24 n = 5 replicates/CRC Conditioned Media). (**e)** Bar graphs presenting numbers of mammospheres formed counted seven days following plating in different media (n = 6 replicates/condition with exceptions IPSI9 n = 3 replicates/EpiCult, IPSI24 n = 5 replicates/CRC Conditioned Media). *p < 0.05. 2way ANOVAs demonstrated an interactive effect between medium and samples accounting for 17.03% of the variance (*p* < 0.0001 F = 193.21 DFn = 21 DFd156) in viability for samples tested in MEGM, EpiC, MammoC and CRC Conditioned Media with sample accounting for 66.54% (p < 0.0001 F = 2264.44 DFn = 7 DFd = 156) and medium 12.33% (*p* < 0.0001 F = 979.30 DFn = 3 DFd = 156) with 7.535% of the variance for samples tested in MEGM, EpiC and MammoC due to an interactive effect (*p* < 0.0001 F = 6.43 DFn = 26 DFd = 204) with sample accounting for 82.71% of the variance (*p* < 0.0001 F = 141.05 DFn = 13 DFd = 204) and medium 0.5213% (*p* < 0.0036 F = 5.78 DFn = 2 DFd = 204). 2way ANOVAs showed an interactive effect accounting for 32.94% of variance (*p* < 0.0001 F = 21.85 DFn = 21 DFd = 157) in mammosphere numbers for samples tested in MEGM, EpiC, MammoC and CRC Conditioned Media with sample accounting for 35.11% (*p* < 0.0001 F = 69.89 DFn = 7 DFd = 157) and medium 20.51% (*p* < 0.001 F = 95.28 DFn = 3 DFd = 157) with 34.01% of the samples tested in MEGM, EpiC and MammoC due to an interactive effect (*p* < 0.001 F = 14.67 DFn = 24 DFd = 195) with sample variance accounting for 44.58% of the variance (p < 0.0001 F = 38.46 Dfn = 12 Dfd = 195) and medium 2.581% (p < 0.0001 F = 13.36 DFn = 2 DFd = 195). Cell viability measured utilizing CellTiter-Glo® 3D with relative viability expressed as CellTiter-Glo luminescent cell viability score. Color coding: Black: Phenol red-free MEGM™. Red: EpiCult™. Blue: MammoCult™. Green: CRC Conditioned Media. *IPSI* ipsilateral non-cancer,*T* tumor cancer.

Ipsilateral high-risk CRC-viable cultures were derived from women with a range of breast cancer types. Advancing age significantly reduced likelihood of initial CRC isolation with no viable samples isolated from the six women ≥ age 70 years (Table 1, Fig. 1a). No effect from exposure to prior neoadjuvant chemotherapy was found. Eight matched invasive tumor samples available were added to the study for comparison. Secondary passage in MEGM was used to expand cultures in serum- and phenol-red-free media in preparation for hormonal response testing. Age did not impact likelihood of growth in MEGM but showed an inverse relationship with measured viability scores (Fig. 1b,c). Because a range of viability was evident in MEGM, two additional mammary-specific media (EpiC and MammoC) along with CRC conditioned media (CM) were tested to determine the extent of media-specific behaviors. Although media differences within samples were identified, viability differences between samples were largely preserved across media (Fig. 1d). Viability was not significantly higher in CRC^CM^ even though this modeled the initial isolation media, albeit without the feeder cell layer. Extent of mammosphere formation was similarly assessed in different media. Samples showed generally consistent behavior across media even with some within sample differences being present (Fig. 1e). A few samples were available for testing across passage. This showed variability between samples was preserved across passage (Suppl. Figure 1a, b). Because the matrix-free scaffold-based nano-culture plates permit both 2D and 3D growth, cell growth patterns were analyzed in each media. The majority of samples showed mixed mammosphere-monolayer cell growth across all media (Suppl. Figure 1c). Only two samples exhibited mammosphere-only growth across all tested media. Phenol-red and serum-free MEGM supported mammosphere growth in all samples. Based on these results, variability in behavior in culture was assessed as a preserved biological property inherent to each sample.

**Table 1:** Table of samples.

Sample code	Cell culture number	Sample type	Breast cancer type	ER	PR	HER2	Elston score	pTNM stage	Sex	Age	*BRCA* mutation status	Second sample from same patient
IPSI1	1067	Non-Cancer Ipsilateral to IDC	IDC	90%	90%	2+	6	pT2pN1c	F	35	No test	T1
IPSI2	1028	Non-Cancer Ipsilateral to IDC	IDC	90%	10%	2+	9	pT3pN3	F	66	Tested negative	T2
IPSI3	987	Non-Cancer Ipsilateral to IDC	IDC	20%	0	negative	8	pT1cpN0	F	67	No test	T3
IPSI4	1015	Non-Cancer Ipsilateral to IDC	IDC	80%	70%	1+	7,8	pT2apN2a	F	60	No test	T4
IPSI5	1018	Non-Cancer Ipsilateral to IDC	IDC	50%	10%	FISH amplified	8	pT1c pN0	F	42	Tested negative	
IPSI6	1111	Non-Cancer Ipsilateral to IDC	IDC	90%	0	negative	9	pT2pN0	F	55	*BRCA1* mutation	
IPSI7	1037	Non-Cancer Ipsilateral to ILC	ILC	90%	0	1+	5	pT1cpN2a	F	63	No test	
IPSI8	1020	Non-Cancer Ipsilateral to IDC	IDC	0	0	3+FISH positive	II of III	ypTxpN1	F	41	Tested negative	
IPSI9	1164	Non-Cancer Ipsilateral to TNBC	TNBC	0	0	negative	9	ypT2pN1	F	44	No test	
IPSI10	978	Non-Cancer Ipsilateral to IDC	IDC	80%	40%	2+equivocal FISH	9	ypT1cpN0(sn)	F	39	Tested negative	T10
IPSI11	973	Non-Cancer Ipsilateral to IDC	IDC	90%	80%	negative	6	pT1pN0	F	49	No test	
IPSI12	1167	Non-Cancer Ipsilateral to IBC	IBC	90%	30%	1+	9	mypT4d pN3a	F	64	No test	
IPSI13	1077	Non-Cancer Ipsilateral to IDC	IDC	80%	80%	2+FISH negative	8	pT1cpN1	F	41	*BRCA2* mutation	T13
IPSI14	1074	Non-Cancer Ipsilateral to IDC	IDC	80%	90%	3+	9	pT1cpN1	F	43	No test	
IPSI15	1000	Non-Cancer Ipsilateral to IDC	IDC	90%	30%	3+	7	ypT0pN1(mi)	F	43	Tested negative	
IPSI16	957	Non-Cancer Ipsilateral to IDC	IDC	80%	80%	1+	5	pT2pN0	F	45	Tested negative	T16
IPSI17	1112	Non-Cancer Ipsilateral to IDC	IDC	80%	10%	1+	5	pT1bpN0	F	55	Tested negative	
IPSI18	1191	Non-Cancer Ipsilateral to IDC	IDC	90%	10%	3+	8	pT2pN0	F	42	Tested negative	
IPSI19	1092	Non-Cancer Ipsilateral to IDC	IDC	90%	90%	3+	6	yT2pN1	F	52	Tested negative	
IPSI20	971	Non-Cancer Ipsilateral to IDC	IDC	90%	80%	negative	6	pT1pN0	F	49	No test	
IPSI21	1120	Non-Cancer Ipsilateral to IDC	IDC	50%	60%	3+	9	ypT0pN1	F	34	Tested negative	
IPSI22	1136	Non-Cancer Ipsilateral to IDC	IDC	0	0	3+	9	pT1cpN0	F	32	Tested negative	
IPSI23	1219	Non-Cancer Ipsilateral to IDC	IDC	na	na	na	na	yTxpNx	F	31	*BRCA1* mutation	
IPSI24	1300	Non-Cancer Ipsilateral to ILC	ILC	95–100%	95–100%	1+	Notting-ham 6	mpT1bpN0(i-)(sn)	F	42	Tested negative	
IPSI25	549	Non-Cancer Ipsilateral to IDC	IDC	95%	70%	1+	6	pT1bpN0	F	46	*BRCA* mutation	
T1	1068	Invasive Breast Cancer	IDC	90%	90%	2+	6	pT2pN1c	F	35	No test	IPSI1
T2	1029	Invasive Breast Cancer	IDC	90%	10%	2+	9	pT3pN3	F	66	Tested negative	IPSI2
T3	988	Invasive Breast Cancer	IDC	20%	0	negative	8	pT1cpN0	F	67	No test	IPSI3
T4	1016	Invasive Breast Cancer	IDC	80%	70%	1+	7,8	pT2apN2a	F	60	No test	IPSI4
T5	1013	Invasive Breast Cancer	IDC	90%	70%	1+	6	pT1cpN0	F	61	No test	
T10	980	Invasive Breast Cancer	IDC	80%	40%	2+equivocal FISH	9	ypT1cpN0(sn)	F	39	Tested negative	IPSI10
T13	1078	Invasive Breast Cancer	IDC	80%	80%	2+FISH negative	8	pT1cpN1	F	41	*BRCA2* mutation	IPSI13
T16	958	Invasive Breast Cancer	IDC	80%	80%	1+	5	pT2pN0	F	45	Tested negative	IPSI16

### Relative expression levels of cell cycle related target genes of E2F transcription factors paralleled relative viability

**Figure 2:**
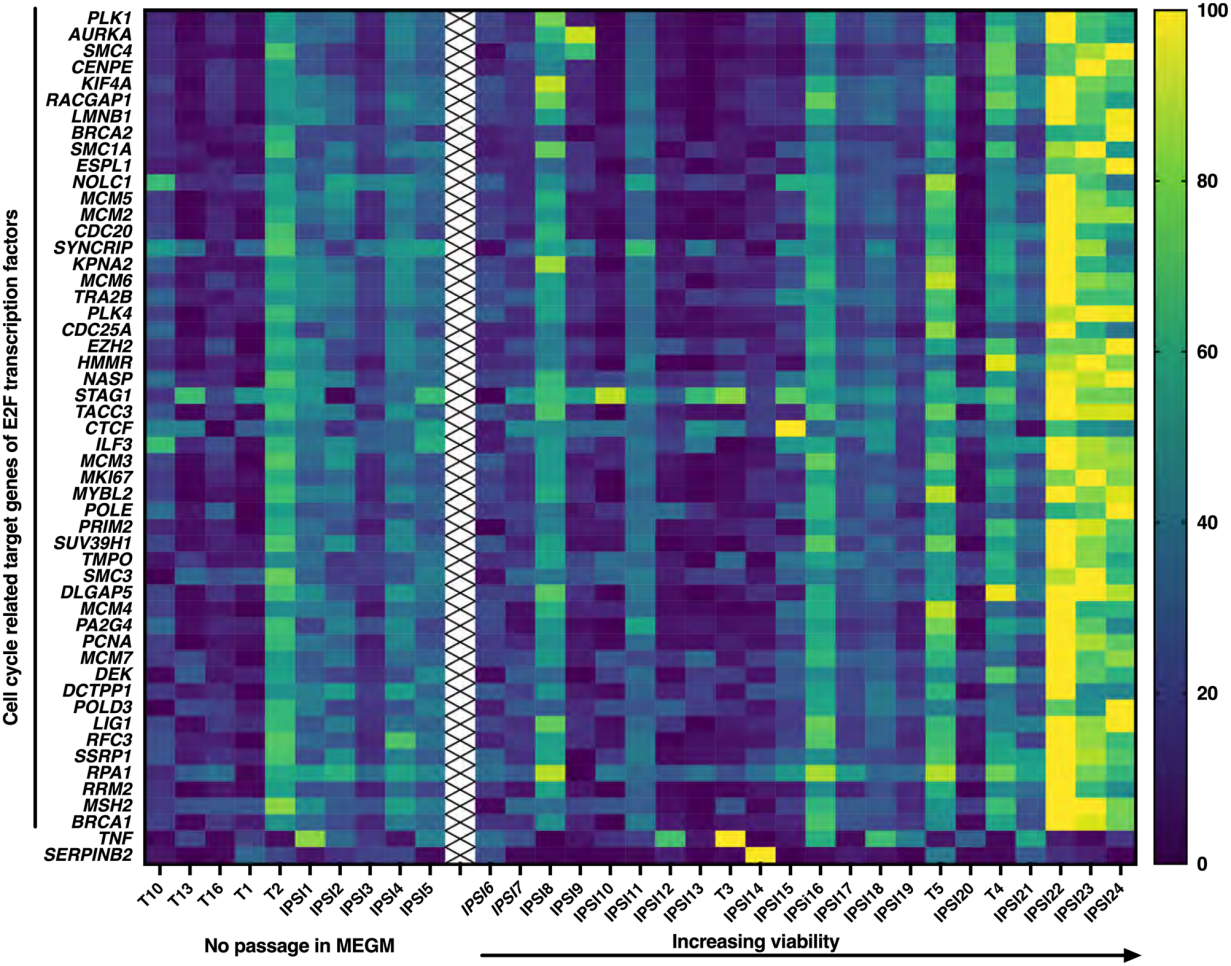
Relative expression levels of cell cycle related target genes of E2F transcription factors. Relative expression levels of cell cycle related target genes of E2F transcription factors (HALLMARK_E2F_TARGETS) are shown with samples arrayed left to right according to increasing viability. Italicized samples (*IPSI6, IPSI7, IPSI8*) were mycoplasma negative on sequence evaluation and grew in MEGM but had no material for biochemical mycoplasma testing and were therefore excluded from presentation of viability and mammosphere numbers. *IPSI* ipsilateral non-cancer, *T* tumor cancer. Color coding: Dark blue to yellow with increasing expression.

Because cell viability was a preserved factor in samples, we asked if expression of genes positively regulating cell proliferation would correlate with relative viability. The HALLMARK_E2F_TARGETS gene set includes cell cycle related target genes of E2F transcription factors. While the highest viability samples showed relative higher expression of many E2F target genes, some samples demonstrated a more selective pattern (Fig. 2). Examples of genes that were recurrently expressed at higher levels in different viable samples included Aurora Kinase A *(AURKA)*, Kinesin Family Member 4A (*KIF4A*) Stromal Antigen 1 (*STAG1*), CCCTC-Binding Factor (*CTCF*), Replication Protein A1 (RPA1), and Tissue Necrosis Factor (TNF). One sample showed higher expression of Serpin Family B Member 2 (*SERPINB2*). This analysis showed that differences in sample viability on secondary passage were linked to transcriptome differences present in the cells upon their initial isolation in CRC.

### Hormonal responsiveness correlated with transcriptomic profile

**Figure 3:**
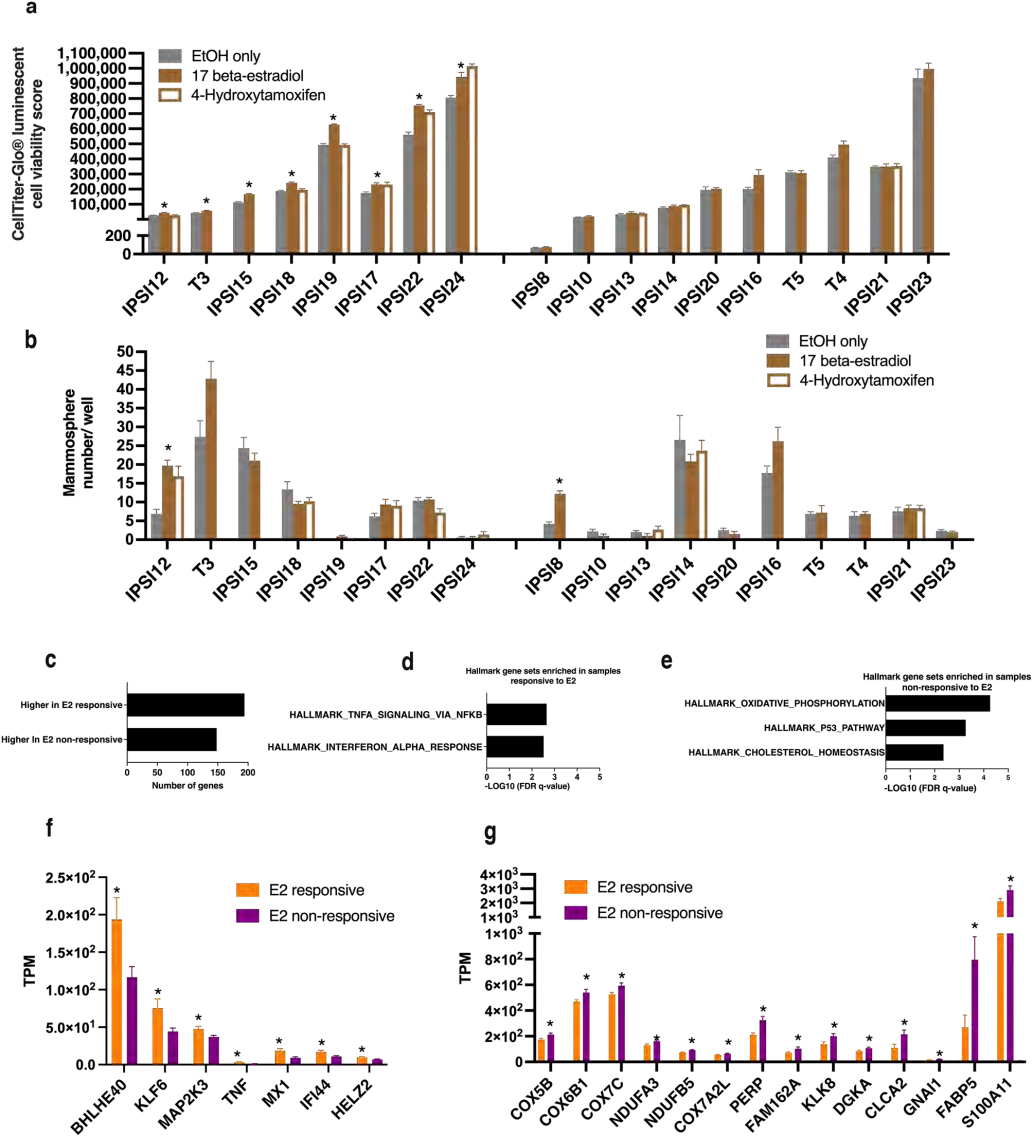
Comparative viability and mammosphere formation following hormonal exposure. (**a**) Bar graph illustrating relative viability following seven-day exposure to 17 beta-estradiol (E2,10 nM), 4-Hydroxytamoxifen (4-OHT, Tamoxifen) (1 µM) or EtOH (n = 6 replicates/condition with exceptions T3 (n = 5/E2), IPSI14 (n = 5/E2), IPSI12(n = 4/4-OHT). (**b**) Bar graph illustrating mammosphere numbers/well following seven-day exposure to 17 beta-estradiol (E2,10 nM), 4-Hydroxytamoxifen (4-OHT, Tamoxifen) (1 µM) or EtOH (n = 6 replicates/condition with exceptions T3 (n = 5/E2), IPSI14 (n = 5/E2), IPSI12(n = 4/4-OHT). (**c**) Bar graph illustrating numbers of genes expressed at significantly higher or lower levels in samples responsive to E2 as compared to samples non-responsive to E2. *q ≤ 0.004, unpaired t tests with Welch correction, Variance assumption: Individual variance for each group, Multiple comparisons: False Discovery Rate, Two-stage step-up (Benjamini, Krieger, and Yekutieli), Desired FDR (Q):1.00%. (**d**) Bar graph showing significantly enriched HALLMARK gene sets for genes expressed at higher levels in E2 respoonsive samples. (**e**) Bar graph showing significantly enriched HALLMARK gene sets for genes expressed at lower levels in E2 responsive samples. (**f**) Bar graph illustrating sample-specific expression levels (TPM) of genes included in HALLMARK_TNF_SIGNALING_VIA_NFKB and HALLMARK_INTERFERON_ALPHA_RESPONSE gene sets. Mean and SEM indicated. (**g**) Bar graph illustrating sample-specific expression levels (TPM) of genes included in HALLMARK_OXIDATIVE_PHOSPHORYLATION, HALLMARK_P53_PATHWAY, and HALLMARK_CHOLESTEROL_HOMEOSTASIS gene sets. Mean and SEM indicated. Cell viability measured utilizing CellTiter-Glo® 3D with relative viability expressed as CellTiter-Glo luminescent cell viability score. *EtOH* Ethanol, *E2* 17 beta-estradiol, *4-OHT* 4-Hydroxytamoxifen, *TPM* transcripts per million, *FDR* false discovery rate, *IPSI* ipsilateral non-cancer, *T* tumor cancer. Color coding: Gray: EtOH. Solid brown: E2. Brown outline: 4-OHT. Orange: E2 responsive. Purple: E2 non-responsive.

To determine if hormonal response would also show a link to the transcriptome, samples were subjected to relative viability measurements in the absence and presence of 17 beta-estradiol (E2) (10 nM) and the ER antagonist 4-hydroxy-tamoxifen (4-OHT) (1 µM) for samples with sufficient numbers of cells for concurrent testing. Of the 18 samples available for concurrent testing, eight showed statistically significant higher viability in E2 (Fig. 3a). Of the nine samples available for concurrent testing in 4-OHT, viability was unchanged in six. Three samples showed higher viability than in the control condition but each had a different pattern. There were no consistent differences between E2 responsive and non-responsive samples in patterns of mammosphere growth (Fig. 3b). One hundred and ninety-four genes were expressed at significantly higher levels and one hundred and forty-eight genes at significantly lower levels in the E2 responsive samples (Fig. 3c). Genes with at least two-fold differences were analyzed by GSEA for significant gene set enrichment. The HALLMARK_TNFA_SIGNALING_VIA_NFKB and HALLMARK_INTERFERON_ALPHA_RESPONSE gene sets were enriched in the E2 responsive samples (Fig. 3d) while HALLMARK_OXIDATIVE_PHOSPHORYLATION, HALLMARK_P53_PATHWAY and HALLMARK_CHOLESTEROL_HOMEOSTASIS gene sets were enriched in the E2 non-responsive samples (Fig. 3e). Differences in expression levels of specific genes within these enriched gene sets between samples responsive and non-responsive to E2 are illustrated using log scales (Fig. 3f,g). In summary, transcriptome differences between samples associated with absence or presence of increased viability in response to E2.

### Genes linked to mammary stem cell formation were expressed at higher levels in samples from women previously exposed to neoadjuvant chemotherapy

**Figure 4:**
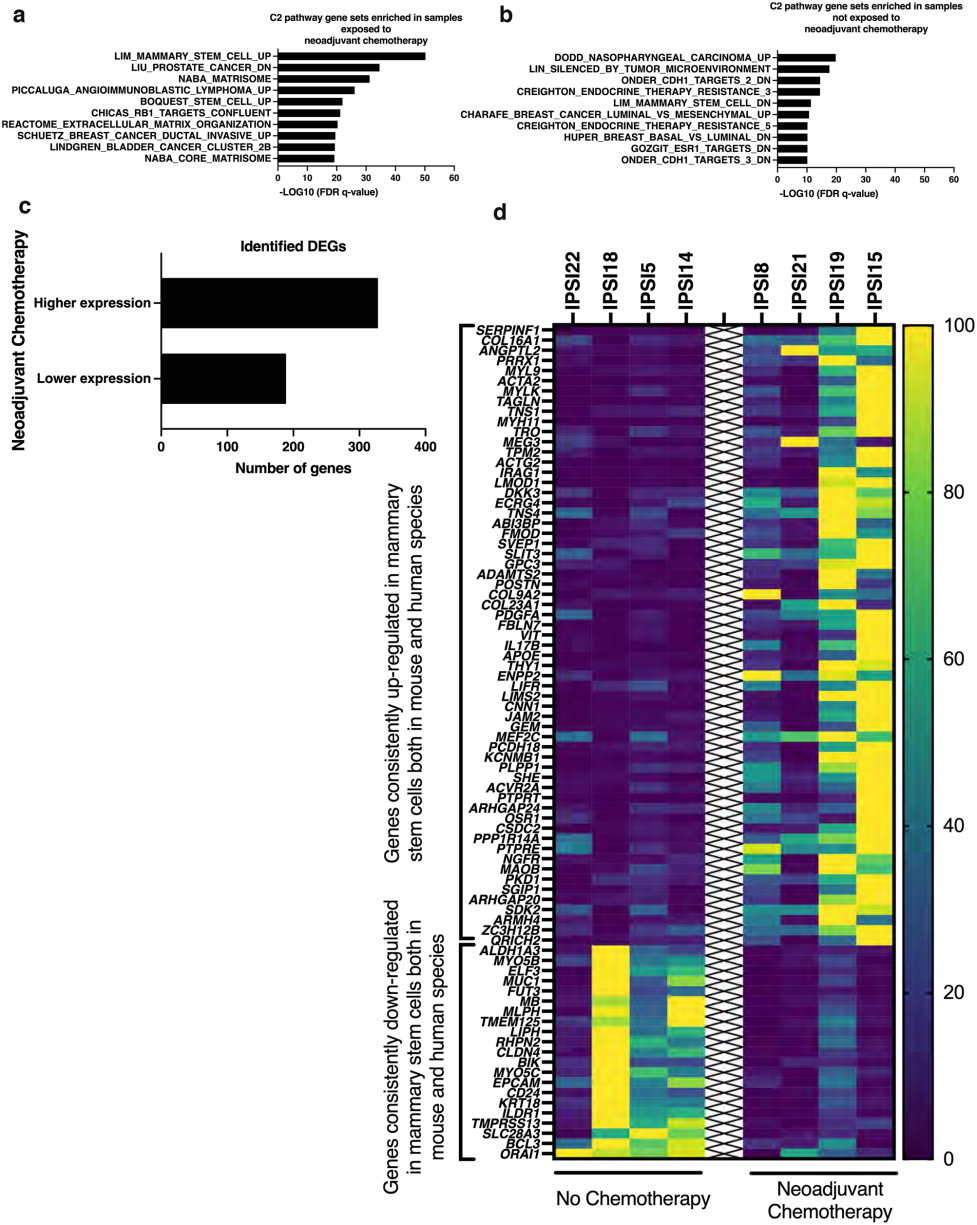
Neoadjuvant chemotherapy exposure associated with gene expression changes linked to mammary stem cell formation in ipsilateral high-risk non-cancer samples. (**a**) Bar graph presenting the top ten C2 gene sets with the lowest significant FDR q-values identified from the MSigDB Collection utilizing genes expressed at significantly higher levels in samples from individuals exposed to neoadjuvant chemotherapy. (**b**) Bar graph presenting the top ten C2 gene sets with the lowest significant FDR q-values identified from the MsigDB Collection utilizing genes expressed at significantly lower levels in samples from individuals exposed to neoadjuvant chemotherapy. (**c**) Bar graph indicating numbers of up-regulated and down-regulated DEGs identified in non-cancer ipsilateral samples from women with ER+/HER2+or HER2+ cancer that were exposed or not exposed to neoadjuvant chemotherapy. (**d**) Heat map illustrating relative expression levels of identified DEGs enriched in LIM_MAMMMARY_STEM_CELL_UP (Genes consistently up-regulated in mammary stem cells both in mouse and human species) and LIM_MAMMMARY_STEM_CELL_DOWN (Genes consistently down-regulated in mammary stem cells both in mouse and human species) in samples from individuals with no chemotherapy exposure and those with neoadjuvant chemotherapy exposure. *DEG* differentially expressed gene at Padj ≤ 0.05, *FDR* false discovery rate, *MsigDB* Molecular Signatures Database v7.5.1, *C2* Curated gene sets. N = 4 no neoadjuvant chemotherapy (aged 40 ± 3 years, mean ± SEM), N = 4 neoadjuvant chemotherapy (aged 42 ± 4 years). Color coding: Dark blue to yellow with increasing expression.

To explore the hypothesis that exposure to neoadjuvant chemotherapy might alter gene expression in the ipsilateral non-cancer samples, we tested the number and character of differentially expressed genes (DEGs) in all ipsilateral samples available from individuals with Erb-B2 Receptor Tyrosine Kinase 2/HER2 positive (HER2+) breast cancer, half of whom had received neoadjuvant chemotherapy. The analysis was limited to this subgroup because this was the only breast cancer subtype that was consistently associated with neoadjuvant chemotherapy administration in our sample set. Limiting the analysis to samples from individuals all of whom had HER2+ invasive breast cancer enabled us to match for any theoretical differences in the ipsilateral high-risk cells that might be associated with development of HER2+ breast cancer. GSEA analyses was performed to identify C2 gene sets enriched in samples from individuals exposed to neoadjuvant chemotherapy and gene sets enriched in samples from individuals not-exposed to chemotherapy. Significantly, the 328 up-regulated genes from samples exposed to neoadjuvant chemotherapy showed enrichment of the LIM_MAMMARY_STEM_CELL_UP gene set while the 189 down-regulated genes, identified due to their higher expression in the absence of neoadjuvant chemotherapy exposure, showed enrichment of the LIM_MAMMARY_STEM_CELL_DOWN gene set (Fig. 4a–c). Relative expression levels of individual mammary stem cell related genes differentially expressed in samples from individuals with and without exposure to neoadjuvant chemotherapy from these two gene sets are shown using a heat map (Fig. 4d). Identification of a pattern of differentially expressed genes in the neoadjuvant-exposed samples that includes genes that are consistently up- and down-regulated in mammary stem cells suggests that mammary stem cell populations could be enriched in the ipsilateral breast tissue of individuals exposed to neoadjuvant chemotherapy.

### Both ipsilateral non-cancer and cancer samples demonstrated aberrant patterns of pregnancy-related gene transcription

**Figure 5:**
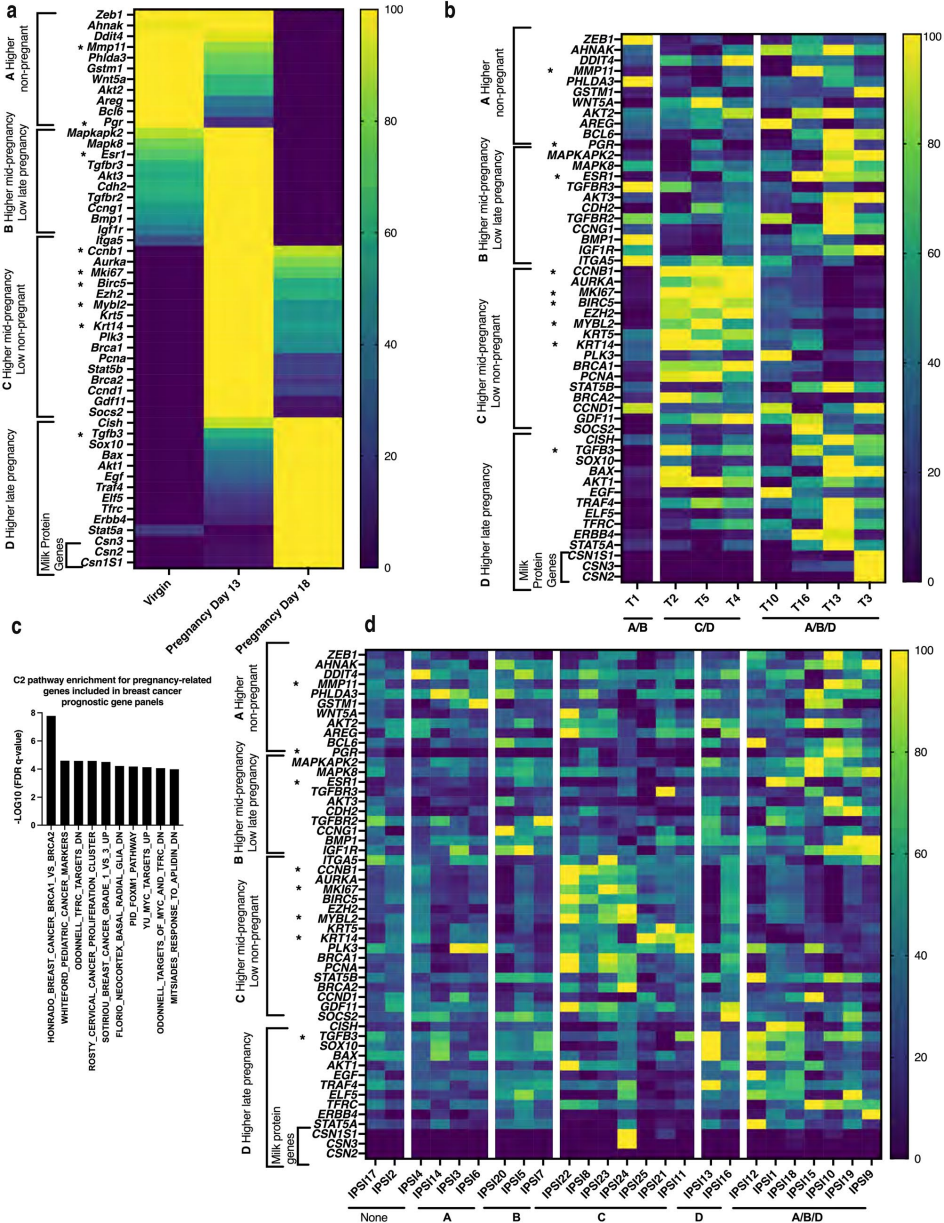
Both ipsilateral high-risk non-cancer and cancer samples demonstrated aberrant expression patterns of pregnancy-related genes. (**a**) Heat map illustrating relative expression patterns of 52 genes known to be expressed in mammary cancer that exhibit pregnancy-stage related changes in gene expression. Values shown from mouse transcriptomic data for virgin, days 13 and 18 pregnancy timepoints (mouse transcriptomic data). A, B, C, and D indicate four stages of distinct patterns of gene expression that change through pregnancy. Asterisks: Genes that are members of established breast cancer prognostic gene profiles. (**b**) Heat map illustrating relative expression patterns of the pregnancy-stage related genes in breast cancer samples sorted by similarity in gene expression pattern. Resemblance to normal patterns (A–D) during pregnancy indicated below x axis. Patterns overlapping more than one stage indicated. (**c**) Bar graph presenting the top 10 C2 gene sets with the lowest significant FDR q-values identified from the MSigDB Collection utilizing pregnancy-stage related genes that are members of established breast cancer prognostic profiles. (**d**) Heat map illustrating relative expression patterns of pregnancy-stage related genes in ipsilateral high-risk non-cancer samples sorted by similarity in gene expression pattern. Correspondance to normal patterns (A–D) during pregnancy indicated below x axis. Patterns overlapping more than one stage indicated. *MsigDB* Molecular Signatures Database v7.5.1, *C2* Curated gene sets. Color coding: Dark blue to yellow with increasing expression.

Pregnancy-associated mammary gland development is characterized by stages of stereotypic gene expression changes regulating normal mammary cell proliferation and differentiation. Abnormal expression patterns of these genes could underlie unregulated cell proliferation associated with breast cancer. To explore this question, sample transcriptomes were queried for adherence to normal pregnancy-associated gene expression patterns. A heat map illustrating changes in gene expression during four different stages of pregnancy (A–D) was developed utilizing mouse transcriptomic data with genes that are members of established breast cancer prognostic profiles indicated by asterisks (Fig. 5a). Relative expression patterns of pregnancy-associated genes in cancer samples were similarly arrayed in a heat map and sorted by expression pattern to determine if there were any links to patterns during pregnancy. Links were found, but patterns invariably overlapped different pregnancy stages, consistent with the notion that cancer cells demonstrate abnormal patterns of genes that normally are not expressed together (Fig. 5b). To address the question of whether or not the limited number of pregnancy-associated genes that are members of an established breast cancer prognostic platform are associated together in established pathways, they were subjected to GSEA analysis (Fig. 5c). This demonstrated that they show significant enrichment in cancer pathways, including breast cancer specific ones, illustrating the potential significance of up-regulated expression in high-risk samples. Finally, relative expression patterns of pregnancy-associated genes in the high-risk samples were arrayed in a heat map and sorted by expression patterns (Fig. 5d). Expression patterns arrayed differently than the cancer samples but still showed links to different pregnancy stages. One-third of the samples showed an overlapping pattern of pregnancy-associated gene expression (A/B/D), similar to that seen in some of the cancer samples. IPSI10 and T10 samples from the same individual showed the same pattern but other cancer samples showed divergent patterns from their ipsilateral counterparts. A portion of samples more closely resembled single pregnancy stage profiles. In summary, a portion of the high-risk cells studied exhibited expression profiles similar to a pregnancy profile.

## Discussion

The overall goal of the study was to study the biology and transcriptional profiles of human mammary epithelial cells at higher than normal risk for breast cancer development. Because women who develop one breast cancer are at risk for secondary breast cancer development, this was approached through isolation of primary mammary epithelial cells from non-cancerous breast tissue obtained from mastectomies performed for treatment of invasive breast cancer. The conditionally reprogrammed cells (CRC) technique was utilized for initial isolation for its efficiency in isolating primary epithelial cells. Transcriptomic studies were performed using cells from this initial isolation condition. Biological studies were performed in mammary-specific media to maintain appropriate differentiation status and provide a suitable environment for testing hormonal response. All studies were performed using low passage number cells to minimize changes that can come with extended passage.

There is reason to consider that the sample diversity identified in this study could be reflective of different risk probabilities for future cancer development. Inspection of the data provides different behavioral and transcriptional profiles for individual samples. This can range significantly. For examples, some samples showed the combination of high viability and E2 responsiveness with a transcriptome including higher E2F target and proliferative mid-pregnancy gene expression that included known breast cancer prognostic genes. Other samples were equivalently E2 responsive but showed an overlapping pattern of different pregnancy stage gene expression. Others showed low viability with or without E2 responsiveness. Without long-term clinical follow-up one cannot define if these different patterns are predictive of different outcomes but they are measurable differences between samples. Only subsets of women develop either primary or secondary breast cancer. The approach utilized here provides additional understanding of at-risk mammary epithelial cell biology that could be further exploited for improvement of secondary breast cancer risk profiling^3,4^.

The study demonstrated associations between behavior and gene expression. Retention of an estrogen growth response correlated with expression of genes enriched in TNF alpha and Interferon alpha signaling pathways. Both of these pathways are established contributors to estrogen signaling^40,41^. Relatively high TNF alpha expression was found in one of the viable samples that lacked high expression of E2F cell cycle target genes. It is not uncommon for cancer cells to rely on a specific growth factors such as TNF alpha for viability^36^. Cancer growth can also be reliant on high expression of specific E2 cell cycle target genes including *AURKA*, *KIF4A*, *STAG1*, *CTCF*, and RPA1, all of which were identified highly expressed in at least one of the at-risk samples studied here^34–39^. One sample with low overall E2F target genes expression demonstrated high levels of *SERPINB* expression, which is associated with maintenance of cell viability^37^. These findings can be interpreted as evidence that high risk cells are poised for future cancer development due to transcriptional changes that favor cell growth and survival. However, whether or not the high-risk cultures could be contaminated with cancer cells has to be considered. This possibility cannot be absolutely excluded but the experimental design included all possible controls for this variable. Ipsilateral samples were taken from breast geographically distinct from the invasive tumor and assessed as non-cancerous on gross examination with mirrored tissue samples from all submitted samples examined histologically for any microscopic evidence of cancer cells. Contaminated samples were excluded from the study.

One can speculate on whether or not the in vitro differences in E2 and 4-OHT response would reflect in vivo response. Anti-hormonals are the mainstay for prevention of secondary cancers for individuals with ER+ cancer, but there is variability in response and resistance can develop^42,43^. Whether or not prospective in vitro testing of hormonal response would ever be clinically useful is an open question. The overall goal for this study, to test a possible platform for evaluating hormonal response of primary mammary epithelial cells and evaluate transcriptome associations was completed. Replication of these results with associated clinical follow-up of anti-hormonal response would be a possible next step.

Neoadjuvant chemotherapy exposure was associated with enrichment of normal mammary gland stem cell gene sets in the high-risk cells^11^. Specific genes relevant to mammary stem cells that were found up-regulated include leukemia inhibitory factor receptor (*LIFR*), reported as promoting breast cancer stem cell renewal^44^ and Thy-1 Cell Surface Antigen (*Thy1*), a gene associated with serial transplantation of mammary epithelial cells^45^. Specific stem-cell related genes that were down-regulated included CD24, a gene which when down-regulated is associated with a breast cancer stem cell phenotype^46^. While we posit that expansion of the mammary stem cell population may be a normal homeostatic restoration of breast tissue post-chemotherapy given chemotherapy can induce lobular atrophy^47^, the character of the surviving stem cells merits additional study. It is known that breast cancer stem cells can survive chemotherapy and the expression pattern identified does have links to both normal and neoplastic mammary stem cells. Whether or not the enriched population found here is reflective of a protective healing response or might provide precursors for future cancer stem cell development is unknown^48^. Hormone receptor expression is a marker for secondary breast cancer risk^49^. The analysis presented here included three hormone receptor positive samples with neoadjuvant chemotherapy exposure (IPSI21, IPSI19, IPSI15) and three hormone receptor positive samples without receipt of neoadjuvant chemotherapy (IPSI18, IPSI5, IPSI14).

Transcriptome data was used to approach a long-standing hypothesis in the field, that is, aberrant expression patterns of genes expressed in normal development might serve as risk factors for cancer generation^50^. Nine of the 52 pregnancy-associated genes in the presented profile are members of at least one established breast cancer prognostic screen^14^. There are pregnancy-related genes known to have clear links to human breast cancer such as *BIRC5*^14^ and *STAT5A*^51^, which were found increased in a subset of samples here. A question we considered is whether or not the patterns found related to different menstrual cycle phases^9^. While the study did include women under the age of 50, differences were found between patterns seen here and the luteal phase patterns. Samples with higher expression of five of the 10 genes expressed during the luteal phase were found but expression was limited to these five genes (*BRCA1, CCNB1, MKI67, BIRC5, EZH2, PCNA*) and the full luteal pattern was not seen. Future studies would be recommended to include menstrual cycle history at the time of collection to directly address this question. Identification of both cancer and high-risk samples with combinations of gene expression that are not found during normal pregnancy development is consistent with the possibility of a link between deregulated expression of pregnancy genes and cancer risk.

From a technical aspect, CRC-technology was a cost-effective approach to develop a collection of high-risk human breast cells that could be effectively exploited for comparison of growth characteristics under different mammary-specific media and hormonal conditions. Use of matrix-free scaffold-based nano-culture plates enabled us to process replicate samples in parallel at reasonably high efficiency for simultaneously scoring of viability and mammosphere growth in different media and under different conditions^14^. Consistent with previous reports that CRC technology supports preservation of stem/progenitor cells^21^, we found uniform ability to form mammospheres upon secondary culture.

In conclusion, at-risk cells preserved behavioral and transcriptome diversity that could reflect different risk profiles, providing a baseline for further development of possible prognostic platforms for breast cancer risk. It is clear that platforms would be challenging to build. For example, they would require clinical validation that might take decades to develop, given the long time frame for breast cancer recurrence. However, utilization of a CRC-based approach would make this feasible as it enables bio-banking of primary breast at-risk cells that can be renewed as needed. This allows them to be available for recurrent experimentation as new technologies and new questions arise over a long-term follow-up study.

Limitations of the study include inadequate sample numbers from men and individuals over age 70. Previously, we used CRC technology to successfully isolate primary squamous cell carcinoma metastatic to salivary gland from individuals aged 70–80^20^, but metastatic epithelial cancer cell and non-cancer breast epithelial cell biology are significantly different and alternative approaches for non-cancerous mammary epithelial cells from aging individuals may be needed^19^. Only one site was utilized due to funding limitations and to facilitate successful handling procedures but future studies could utilize different sites to help expand diversity. Samples from women with ER+/HER2+ breast cancer were relatively over-represented (22%) and samples from triple-negative under-represented (7%) compared to U.S. population frequency (13.4% and 13%, respectively)^52^. The distribution towards successful isolation from younger age individuals contributed to this under-representation because the majority of triple negative samples submitted were from older women. Because the study was designed to characterize in vitro cell behavior at the lowest passage number possible to limit media-induced behavioral and gene expression changes^19^, few samples were studied across different passages. Passage five was the highest passage number evaluated. Because the study focused on primary cell behavior, establishment of cell lines was not attempted. A limitation was that it was not possible to obtain primary cell cultures of the same sample at the same passage at different points in time so each sample could be studied in temporally distinct experiments, rather than with multiple replicates at a single timepoint as was performed here. However, while it was not feasible to test all samples in more than one experiment, a subset of samples that passaged well were repetitively studied in two or three different experiments across passage. Results showed general consistency across the individual experiments (Supplementary Figure 1). Funding was insufficient to include a limiting dilution analysis for mammosphere formation. Protein expression validation was also not able to be included, however, in previous studies we have validated concordance between quantitative and specific RNA and qualitative protein measurements^19,20^. The pregnancy gene expression profile was developed from RNAseq of whole mammary gland tissue, which would also contain populations of stromal and other cells that could influence gene expression levels shown^53^. To help address this issue, the pregnancy-related gene profile was limited to genes known to be expressed in mammary adenocarcinoma cell lines. Although both ipsilateral and cancer sample tissues were evaluated to confirm histological identity, the possibility remains that either an ipsilateral sample would contain some infiltrating tumor cells or, conversely that a tumor sample might contain non-cancerous cells that could affect results.

## Methods

### Research involving human participants

This study was approved by the Institutional Review Board (IRB) of the Office of Research Oversight/Regulatory Affairs, Georgetown University. It was determined to impose minimal risk on participants. Informed consent was obtained. All research was performed in accordance with relevant guidelines/regulations. This included deidentification of all samples and medical records with assignment of unique GUMC identifier.

### Human sample acquisition and RNA sequencing

Experiments were designed to ethically collect de-identified human breast tissue from living individuals with a diagnosis of breast cancer or at high-risk for breast cancer, with biospecimens obtained from surgically excised tissue not needed for pathologic diagnosis by a board-certified pathologist at room temperature, stabilized by immersion in CRC-compatible media, with mammary epithelial cells isolated from minced tissue incubated in media containing a collagenase/hyaluronidase/dispase solution followed by culture at 37 °C utilizing CRC conditions^18–21^, and viably stored in liquid nitrogen (− 196 °C) for < 1 to 4 years. Ipsilateral non-cancerous breast tissue (volume 2 cm^3^) were procured from grossly unremarkable fibroadipose mammary parenchyma, at least 6–8 cm away from any tumor. Breast cancer tissue (volume 2 cm^3^) was included for comparative evaluation when consent provided. Tissue was processed into two “mirrored” (volume 0.5^3^–1 cm^3^) samples: one for CRC technology and one for formalin-fixation. Deidentified pathology reports provided clinical and pathology diagnoses including age, breast cancer subtype, pathologic stage, *BRCA* gene mutation status, lymph node or other metastasis, and history of neoadjuvant chemotherapy. Numbers of ipsilateral tissue samples for acquisition were determined prior to the experiment (n = 50). Fifty-six samples were obtained. The only study inclusion factor was that the individual was undergoing mastectomy for breast cancer treatment. Final classification of samples as invasive tumor or non-cancer was determined after review of H&E sections of the formalin-fixed “mirrored” tissue by a board-certified pathologist. One ipsilateral non-cancer specimen was re-classified as invasive tumor. All samples very processed equivalently and no ipsilateral or invasive tumor submitted sample was excluded from the study. Unique Georgetown University Medical Center (GUMC) primary cell culture identifiers were assigned following initial isolation^20^. Epithelial cell growth was separated from the underlying fibroblast feeder layer by differential trypsin treatment, divided, and viably frozen as Passage 0 (P0). The majority (83%) of ipsilateral samples yield 4 tubes with a minority yielding only two tubes. RNA sequencing (RNAseq) was performed on P0 tubes when > 2 tubes available with remaining samples being secondarily passaged using CRC technology and a P1 tube used. Total RNA isolated from cell pellets (RNeasy Mini Kit, Qiagen, Gaithersburg, MD) was quantified, analyzed for quality (Nanodrop, ThermoFisher Scientific, Wilmington, DE Bioanalyzer 2100, Agilent Technologies, Santa Clara, CA). For 86% of the ipsilateral samples, 1 μg ribosome-depleted RNA was used for stranded-specific paired-end library preparation (Illumina TruSeq Stranded mRNA Library Preparation Kit (polyA cDNA synthesis) (San Diego, CA) and sequenced (Illumina HiSeq4000 machine, 150 bp pair-ended lane, minimum reads ≥ 100 M per sample). For 14% of the ipsilateral samples, 1 μg ribosome-depleted RNA was used for shotgun library construction (200 bp insert) and sequenced (Illumina HiSeq2000, 91 bp pair-ended lane generating 2 Gb/sample)^20^. Quality check (FastQC), quality trimming (Trim galore) and alignment (STAR) were performed^54^ according to library preparation method with batch effect normalization^55^. Normalized expression levels were estimated by means of transcripts per million (TPM) using RSEM^56^. Sequences were mapped to a merged human + mouse genome file (HG38 + mm10) to assess for mouse fibroblast feeder contamination of epithelial cell pellets. Samples with > 10% mouse sequence contamination were excluded from further analysis. Sequences were then mapped to a mycoplasma genome file for detection of mycoplasma contamination^29^. Samples with detectable mycoplasma sequences were excluded from further analysis. Sample numbers retained following screening for human and mycoplasma sequences and confirmed diagnosis of invasive tumor in the individual from whom samples were obtained were ipsilateral (IPSI) n = 25 (97% ± 0.44 (mean ± SEM) human sequence) and invasive tumor (T) n = 8 (97% ± 0.86 human sequence) (Table 1). Differentially expressed genes were identified using DESeq2^57^. Genes were considered statistically significantly differentially expressed when Padj < 0.05.

### Passage in MEGM, comparative culture in MEGM, EpiCult™, MammoCult™, CRC^CM^ , and assessment of hormonal response

Cells were thawed from CRC pellet with the exception of one ipsilateral sample that was trypsinized (Trypsin-EDTA 0.05%, Thermofisher Scientific Waltham, MA) directly from secondary CRC culture. Cells were collected, centrifuged at 1000xRPM, reconstituted in serum-free, phenol-red free MEGM (Lonza) and counted using a TC20™ Automated Cell Counter (Bio-Rad Laboratories, Hercules, CA). Cells were assessed for expansion in MEGM for one passage with media renewal every third day in T25 flasks (Nest Biotechnology, Jiangsu, China) at 37 °C in 5% CO2 incubator with a goal of 80% confluency to enable sufficient cell numbers for comparative media and hormonal growth studies. Biochemical mycoplasma testing was performed following MEGM passage (MycoAlertTM Mycoplasma Detection Kit, Lonza). Cultures testing positive or with inadequate testing material were excluded from further analyses. When target confluency was reached, cell cultures were trypsinized (phenol-red-free, TrypLE Express Enzyme (1x) trypsin,ThermoFisher Scientific cat no: 12604021), collected, centrifuged at 1000xRPMs, reconstituted in MEGM, counted, viability estimated (0.4% trypan blue in saline dye exclusion) and seeded into 3D NanoCulture Low-Binding Micro Honeycomb 96 well-plates (ORGANOGENIX, Japan)^25^ (10 × 10^3^ cells/well, 0.1 mL medium/well, n = 6 replicates/condition/passage number with exceptions for insufficient cell numbers (IPSI9, n = 3 replicates (EpiC); IPSI24 (CRC Conditioned Media), T3, IPSI14 n = 5 replicates (E2), IPSI12 n = 4 replicates (4-OHT). For each sample, multiple replicates of independently plated and processed cells at a single passage within a single experiment were performed to limit possible passage induced variability. Cell viability and mammosphere formation after seven days growth were determined in three different mammary specific media; MEGM, EpiCult™-C Human Media (STEMCELL Technologies) supplemented with 10 ng/mL EGF (cat no: PHG0311) and bFGF (cat no: PHG0261) (Thermofischer Scientific) and 0.48 µg/mL Hydrocortisone (STEMCELL Technologies, MammoCult™ Human Media (MammoC) (STEMCELL Technologies) supplemented with 4 µg/mL Heparin (STEMCELL Technologies) and 0.48 µg/mL Hydrocortisone, and Conditioned Media (CM)^20^. Media was renewed daily by removing 0.05 mL media and replacing it with 0.05 mL fresh media. Hormone response was assessed in serum-free, phenol-red free, MEGM. 17β-Estradiol (E2) (cat no:50–28-2) and 4-Hydroxytamoxifen (4-OHT) (98% Z isomer, cat no: 68047–06-3) (Sigma Aldrich (St. Louis MO). Stock solutions were prepared in pure Ethanol (EtOH), stored at 20 °C in 1 mL aliquots and diluted in MEGM for use in cell culture. Cell cultures were treated with 10 nM E2/0.1% EtOH (1 mM), 1 µM 4-OHT/0.1% EtOH (1 mM) or vehicle (EtOH) alone for seven days with daily renewal of media and treatment.

### Mammosphere and cell viability measurements

Cell aggregates were counted as mammospheres if they were equal or greater than 100 μm in diameter^58,59^. After seven days of culture at 37 °C in a 5% CO2 incubator, each well was imaged in entirety using phase contrast microscopy (4x, EVOS FL Cell Imaging System, Life Technologies, Paisley, UK), numbers of mammospheres counted in each well, and cell growth patterns recorded as monolayer only, mammosphere only, or mixed monolayer and mammosphere. Cell viability was measured (CellTiter-Glo® 3D, Promega Corporation, Madison, WI, cat no: G9681) by removing all media, adding reagent to each well, then shaking plates (five minutes). Luminescence was recorded after 30 min (room temperature) with the signal measured at 1.0.s increments using a VICTOR Multilabel Plate Reader (PerkinElmer, Waltham, MA).

### Gene set enrichment analyses and data visualizations

Mycoplasma-free samples on sequence analysis [ipsilateral (IPSI, n = 25) and invasive tumor (T, n = 8) samples placed into initial MEGM culture were queried for TPM values of HALLMARK_E2F_TARGETS gene set (Molecular Signatures Database v7.4 C1, accessed July 2021)^31,32^. TPM values were normalized and relative expression levels visualized (Heat Map, GraphPad Prism 9.3.1, GraphPad Software, LLC. San Diego, CA). To investigate possible gene expression changes induced by neoadjuvant chemotherapy in ipsilateral breast of individuals with ER/PR/HER2+ or HER2+ invasive breast cancer, DEGs^57^ were analyzed in samples from individuals that received neoadjuvant chemotherapy prior to sample acquisition (n = 4) as compared to samples from individuals that did not (n = 4). Significantly up- and down-regulated DEGs were analyzed separately (C2 gene sets, GSEA)^31,33^. Enrichment in gene sets related to mammary epithelial stem cells were identified for both (accessed July 2021) (FDR q-value < 0.05). TPM values of the genes found enriched in these two gene sets were normalized and relative expression levels visualized (Heat Map, GraphPad Prism 9.3.1). All cultures with sufficient numbers of cells for this evaluation were tested for significant differences in viability and mammosphere formation in the presence of E2 (n = 18). As a comparator, viability in 4-OHT was examined in the nine samples that had additional sufficient cells for this evaluation. The transcriptomes of samples with significantly higher viability in E2 were compared to those with equivalent viability to identify genes expressed at significantly different levels (Multiple unpaired t tests with Welch correction, Two-stage step-up, Graphpad Prism, q ≤ 0.004 considered statistically significant). Significantly differently up- and down-regulated genes were analyzed separately for enrichment in HALLMARK gene sets (GSEA, accessed February 2022, FDR q-value < 0.05). TPM values of genes found in enriched gene sets were visualized on bar graphs (GraphPad Prism 9.3.1).

### Pregnancy-linked breast cancer risk genes

A heat map illustrating changes in relative gene expression in the mammary gland during pregnancy was generated using downloaded data generated from virgin and pregnant 2-month-old mice (GSE70440)^8^ (GraphPad Prism 9.3.1). Four patterns (A–D) of gene expression changes during pregnancy were identified based on their relative expression levels in non-pregnant (virgin), day 13 and day 18 pregnancy. Pregnancy-related genes that are included in at least one validated breast cancer prognosis platform were identified^26^ and subjected to GSEA analysis for C2 gene set enrichment. Heat maps of pregnancy-related genes were generated separately for invasive tumor (T) and ipsilateral (IPSI) samples (GraphPad Prism 9.3.1). Gene expression patterns of the individual samples were visually sorted by similarity. Three different patterns were recognized in the invasive tumor samples and six different patterns in the ipsilateral samples. These patterns were then compared to the four pregnancy-related patterns. Patterns were assigned to a single pregnancy-stage-related pattern or overlapping pregnancy-stage-related patterns dependent upon the gene expression data.

### Analyses for chromosomal deletions and amplifications

Transcriptomes from four mycoplasma-free validated invasive tumor/ipsilateral pairs were available to assess for known breast cancer-related chromosomal deletions/amplifications (Suppl. Table 1)^57^. DEGs between T4/IPSI4, T13/IPSI13, T3/IPSI3 and T10/IPSI10 were identified using DESeq2 (Padj < 0.05 considered statistically significant). Chromosomal regions of differentially expressed genes were identified using C1:positional gene sets (Molecular Signatures Database v7.4 C1, nominal *p* value < 1%) (accessed August 2019)^31,33^. cBioPortal (accessed February 2021, nine different human breast cancer databases queried) was used to identify specific percentages of chromosomal region/gene amplifications/deletions in the paired samples^60–62^.

### Statistical analyses

For RNAseq, quality check (FastQC), quality trimming (Trim galore) and alignment (STAR) were performed^54^ according to library preparation method and batch effect normalization conducted^55^. Normalized expression levels were estimated by means of transcripts per million (TPM) using RSEM^56^ and differentially expressed genes were identified using DESeq2 (*padj* ≤ 0.05 considered statistically significant)^57^. Mean ± standard error of the mean (SEM) for patient ages, viability measurements and mammosphere counts were calculated (GraphPad Prism 9.3.1). Ordinary one-way ANOVA with Brown-Forsythe test and Sidak’s multiple comparisons test was used to compare probability of CRC isolation by age and neoadjuvant chemotherapy exposure (*p* ≤ 0.05 considered statistically significant, GraphPad Prism 9.3.1). Fisher’s exact, two-tailed was used to compare probability of MEGM passage in cancer versus non-cancer cells (*p* ≤ 0.05 considered statistically significant, GraphPad Prism 9.3.1). 2way ANOVA was used to analyze for statistically significant interactions between samples and media for viability and mammosphere numbers for groups with three and four media (*p* ≤ 0.05 considered statistically significant, DF values reported in figure legend 1, GraphPad Prism 9.3.1). Multiple unpaired t-tests using FDR approach (Two-stage step-up method of Benjamin, Krieger and Yekutieli, GraphPad Prism 9.3.1) was used to examine for statistically significant differences for two media comparison of viability and mammosphere numbers, differences in hormonal response for viability, mammosphere numbers and TPM values for hormonal response (*p* < 0.05 statistically significant, t, df values reported in figure legend 3, GraphPad Prism 9.3.1). Simple linear regressions were conducted to analyze for significant relationships between MEGM viability and patient age (*p* ≤ 0.05 statistically significant, GraphPad Prism 9.3.1). Regression equations, *p* and R squared values presented on regression scatter plot graph (Fig. 1c). Scatter plots, stacked bar graphs, bar graphs and heat maps were prepared in GraphPad Prism 9.3.1.

### Ethical approval

This study was approved by the Institutional Review Board (IRB) of the Office of Research Oversight/Regulatory Affairs, Georgetown University.

### Informed consent

Informed consent was obtained prior to sample acquisition.

## Supplementary Information

Supplementary Information.

## Data Availability

The data discussed in this publication were deposited in NCBI's Gene Expression Omnibus^63^, accessible through GEO Series accession number GSE 185314 (https://www.ncbi.nlm.nih.gov/geo/query/acc.cgi?acc=GSE185314).
