# Supplementary material for: Characterization of transcriptome diversity and in vitro behavior of primary human high-risk breast cells
Source: Sci Rep. 2022 Apr 22;12:6159. doi: 10.1038/s41598-022-10246-4 (PMC9033878; doi:10.1038/s41598-022-10246-4)
Supplement: Supplementary file 1 — Supplementary Information. [file 41598_2022_10246_MOESM1_ESM.pdf]

Supplementary Figure 1.

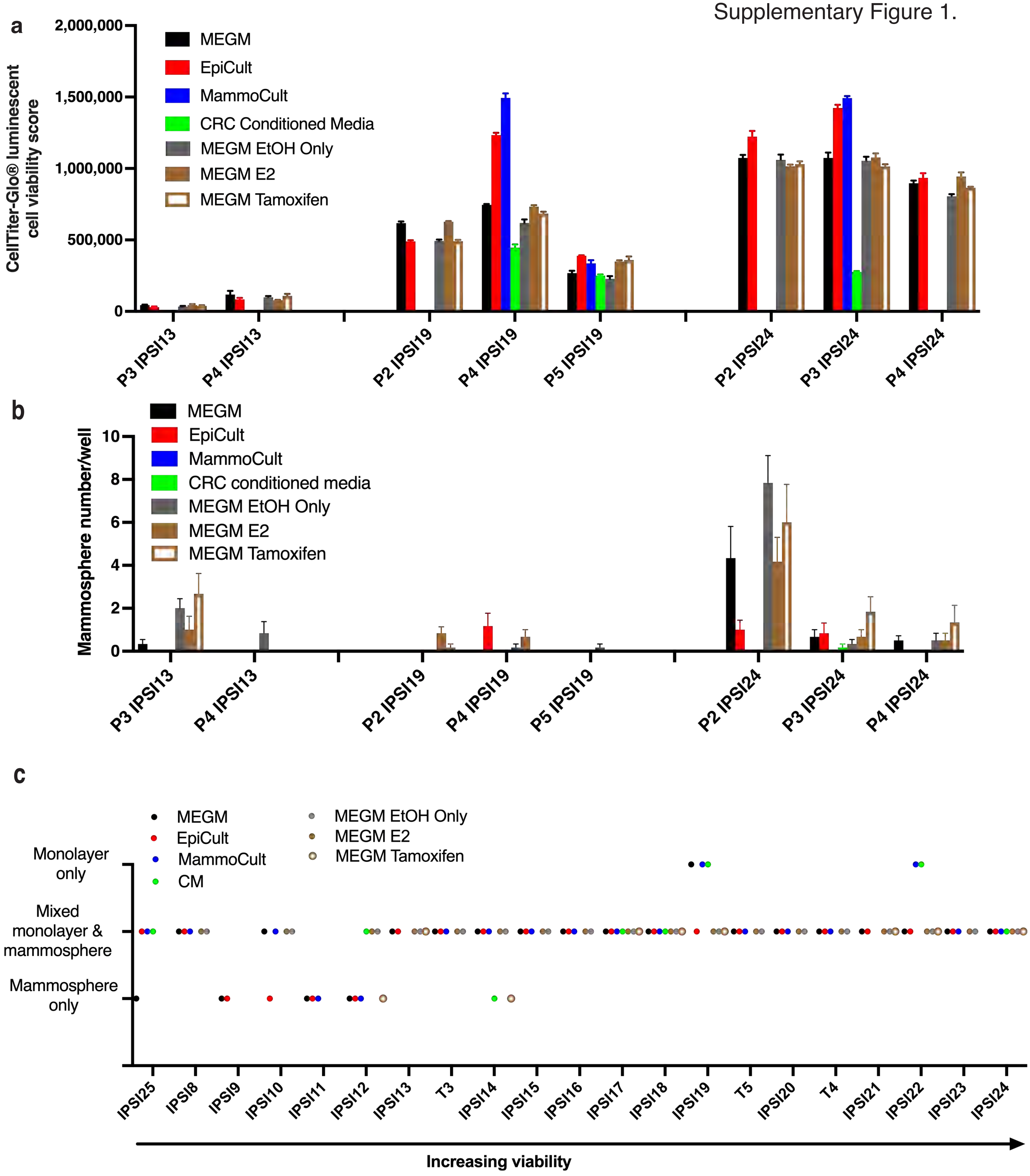

Supplementary Figure 1.

a. Viability comparison for all samples tested across different passages (IPSI13, IPSI19, IPSI24) (n=6 replicates/condition). **b.** Mammosphere number comparison for all samples tested across different passages (IPSI13, IPSI19, IPSI24) (n=6 replicates/condition). **c.** Scatter plot of cell growth patterns of all samples assessed for viability and mammosphere formation presented in Figure 1 arrayed by increasing viability. Cell viability measured utilizing CellTiter-Glo® 3D with relative viability expressed as CellTiter-Glo luminescent cell viability score. Color coding: Black: Phenol red-free MEGM™. Red: EpiCult™. Blue: MammoCult™. Green: CRC Conditioned Media. Gray: MEGM with EtOH vehicle control. Solid brown: MEGM with 17 beta-estradiol (E2) (10nM). Brown outline: MEGM 4-Hydroxytamoxifen (4-OHT) (1μM). P: Passage.

Supplementary Table 2.

Cancer/ Non-cancer Sample Pair	Chromosomal Regions of Gene Sets Significantly Enriched in Cancer Sample (in order of nominal *p* value, all <1%)[1]	Representative genes identified as expressed at significantly higher levels in cancer sample (padj<0.05)[1,2]	Range of amplification percentage reported in human breast cancers[3]	Chromosomal Regions of Gene Sets Significantly Enriched in Non-Cancer Sample (in order of nominal *p* value, all <1%)[1]	Representative genes identified as expressed at significantly lower levels in cancer sample (padj<0.05)[1,2]	Range of deep deletion percentage reported in human breast cancers[3]
T4/IPSI4	CHR1P31	*JAK1*	0.16-1.85%	CHR19 Q13	*UBE2M*	0.13-1.27%
		FUBP1	0.1-1.59%		*TRIM28*	0.13-1.27%
	CHR2Q31	*H3F3AP4*	0.41-10.55%		*SLC27A5*	0.13-1.27%
		PDK1	0.26-2.11%	CHR19P13	*CENPBD1P1*	0.13-1.27%
		NFE2L2	0.26-0.93%		*DOCK6*	0-4.22%
	CHR15Q21	*MYO5A*	0.69-1.57%	CHR11Q13	*MEN1*	0-3.38%
		USP8	0.48-1.27%	CHR16P13	*CIITA*	0-0.14%
		TCF12	0-3.8%	CHR9Q34	*CDK9*	0-0.42%
	CHR2Q32	*PMS1*	0.42-1.35%	CHR17Q25	*TBCD*	0-1.27%
	CHR12Q23	*SLC25A3*	0.28-1.39%	CHR7P22	*FAM20C*	0.13-0.93%
	CHR6Q22	*PTPRK*	1.16-6.48%	CHR3P21	*BAP1*	0.1-1.69%
		GOPC	0.93-3.24%		*MST1R*	0.18-1.69%
	CHR12Q21	*BTG1*	0.97-6.02%	CHR16P11	*SLX1A*	0-0.05%
	CHR16Q12	*CYLD*	0-1.39%	CHR20P13	*RBCK1*	0.26-0.86%
	CHR5Q33	*CD74*	0-0.93%		*TRIB3*	0.26-0.86%
	CHR13Q14	*RB1*	0-0.46%	CHR22Q12	*MCM5*	0-0.14%
	CHR7Q31	*MET*	0-2.31%	CHR16Q22	*HAS3*	0.74-1.67%
		POT1	0.39-6.94%		*TERF2*	0.61-1.48%
	CHR18P11	*YES1*	0.47-1.24%	CHR22Q13	*PARVB*	0.51-2.53%
	CHR8Q22	*UBR5*	9.77-21.45%	CHRXQ28	*VBP1*	0.28-0.49%
	CHR4Q31	*FBXW7*	0.1-3.7%		*BRCC3*	0.28-0.49%
		SETD7	0.12-2.53%			
	CHR11Q23	*BCL9L*	0.25-3.8%			
		DDX6	0-4.22%			
		ARHGEF12	0-1.69%			
		PAFAH1B2	0-1.69%			
		PCSK7	0-2.53%			
		SDHD	0.13-1.59%			
	CHR18Q12	*ELP2*	0.46-2.53%			
		ZNF24	0.28-0.93%			

	CHR6P12	*DST*	0.9-4.63%			
T13/IPSI13	CHR11Q22	*YAP1*	0.28-2.56%	CHR19P13	*INSR*	0-5.91%
		ATM	0-0.56%		*SMARCA4*	0.5-3.38%
	CHR7Q31	*POT1*	0.39-6.94%		*DNM2*	0-2.95%
	CHR2Q32	*STAT1*	0.84-2.31%		*CARM1*	0.12-2.95%
	CHR7P14	*GGCT*	0.74-6.33%	CHR16P13	*DNAJA3*	0-0.42%
	CHR12Q21	*CSRP2*	0.9-3.7%	CHR17Q25	*METRNL*	0-1.27%
	CHR5P13	*DROSHA*	0.19-2.72%	CHR22Q1	*CRYBB2P1*	0-0.97%
	CHR15Q21	*USP8*	0.51-1.27%	CHR16P11	*SLX1A*	0-0.05%
		TCF12	0-3.8%	CHR11Q13	*ATG16L2*	0-0.25%
	CHR1P22	*RPL5*	0.46-1.1%	CHR11Q24	*UBASH3B*	0.23-1.35%
	CHR13Q14	*RB1*	0-0.46%	CHR7Q36	*PRKAG2*	0-0.23%
		LCP1	0-0.93%	CHR3P21	*BAP1*	0.1-1.69%
	CHR6Q25	*EZR*	0.46-2.95%		*MST1*	0.18-1.27%
		ARID1B	0-13.5%		*MST1R*	0.18-1.69%
	CHR2P16	*FBXO11*	0-1.2%	CHR8Q24	*KIFC2*	0-0.42%
		MSH6	0-1.2%	CHR11Q12	*FAM111A*	0-2.53%
				CHR11P11	*DDB2*	0-0.12%
T3/IPSI3	CHR2Q24	*ACVR1*	0.7-1.85%	CHR19P13	*SMARCA4*	0.5-3.38%
	CHR8P11	*ZNF703*	11.59-18.57%		*TCF3*	0.07-6.63%
		NSD3	10.04-14.71%		*CARM1*	0.12-2.95%
	CHR1P31	*JAK1*	0.16-1.85%		*CALR*	0-1.69%
	CHR6P22	*DEK*	1.41-3.38%		*DNMT1*	0-2.95%
		HIST1H1C	0.36-8.57%		*KEAP1*	0-2.95%
		HIST1H2AC	0.83-14.77%		*FSTL3*	0.09-7.59%
		HIST1H2BD	0.31-14.77%	CHR22Q11	*CRKL*	0.05-0.84%
		TRIM27	0.13-2.11%		*SMARCB1*	0-0.42%
		HIST1H2BK	0.51-5.06%		*HIRA*	0.09-0.84%
	CHR3Q13	*CBLB*	0.51-4.63%		*BCR*	0-0.42%
		GSK3B	0-1.23%		*LZTR1*	0.05-0.84%
	CHR10Q21	*ARID5B*	0.63-10.97%	CHR9Q34	*TRAF2*	0-1.27%
		CCDC6	0.97-13.5%	CHR17P13	*YWHAE*	0.46-1.27%
	CHR9Q21	*GNAQ*	0.21-0.56%		*TP53*	0.23-5.91%
		NTRK2	0.1-0.93%		*RABEP1*	0.23-0.86%
				CHR16Q22	*CDH1*	0.36-2.04%
					CBFB	0-1.57%
T10/IPSI10	CHR9Q22	*TGFBR1*	0-0.28%	CHR12P13	*KDM5A*	0-0.46%

	CHR1P34	*SFPQ*	0.46-1.27%	CHR11Q22	*CDKN1B*	0-1.27%
	CHR11Q24	*ETS1*	0-3.24%		*PTPN6*	0-0.42%
		CHEK1	0-0.32%		*YAP1*	0-3.38%
	CHR6Q25	*EZR*	0.46-2.95%	CHR6P21	*TAP1*	0-0.19%
					TFEB	0-0.28%
					MDC1	0-0.19%

[1] https://www.gsea-msigdb.org/gsea/msigdb/index.jsp#citing_msigdb accessed August 2019 (C1 Positional Gene Sets). Subramanian, Tamayo, et al. (2005, PNAS 102, 15545-15550), Liberzon, et al. (2011, Bionformatics). Alternatives and additional info: http://software.broadinstitute.org/gsea/msigdb/collection_details.jsp#C1 de Preter et al. Positional gene enrichment analysis of gene sets for high-resolution identification of overrepresented chromosomal regions. Nucleic Acids Research, 2008, Vol. 36, No. 7 e43 doi:10.1093/nar/gkn114

[2] See Methods: [1]Kang, Keunsoo. Dankook University. Methods. Batch effect between RNA-seq samples in different batches are corrected using RUVSeq (Risso D. et al. (2014) Normalization of RNA-seq data using factor analysis of control genes or samples. Nat. Biotechnol., 32, 896-902). Normalized expression levels are estimated by means of transcripts per million (TPM) using RSEM (Li B. and Dewey C. N. (2011) RSEM: accurate transcript quantification from RNA-Seq data with or without a reference genome. Bioinformatics, 12, 323) Differentially expressed genes are identified using DESeq2 (Love M. I. et al. (2014) Moderated estimation of fold change and dispersion for RNA-seq data with DESeq2. Genome Biol., 15, 550) Genes are considered statistically significantly differentially expressed with Padj <0.05. As indicated, one sample set analyzed (537/536) was later determined to be mycoplasma contaminated at time of initial CRC isolation.

[3] cbioportal.org accessed February 7-8, 2021. Databases queried: Metastatic Breast Cancer (INSERM, PLoS Med 2016), The Metastatic Breast Cancer Project (Provisional, February 2020), Breast Invasive Carcinoma (TCGA, Firehose Legacy), Breast Invasive Carcinoma (TCGA, Cell 2015), Breast Cancer (METABRIC, Nature 2012 & Nat Commun 2016), Breast Invasive Carcinoma (TCGA, PanCancer Atlas), Breast Invasive Carcinoma (TCGA, Nature 2012), Breast Cancer (MSKCC, NPJ Breast Cancer 2019), Breast Cancer (MSK, Nature Cancer 2020)
